# The performance of ChatGPT and Bing on a computerized adaptive test of verbal intelligence

**DOI:** 10.1371/journal.pone.0307097

**Published:** 2024-07-25

**Authors:** Balázs Klein, Kristof Kovacs

**Affiliations:** 1 Testar Ltd., Budapest, Hungary; 2 ELTE Eotvos Lorand University, Budapest, Hungary; University of Maribor Faculty of Health Sciences: Univerza v Mariboru Fakulteta za Zdravstvene Vede, SLOVENIA

## Abstract

We administered a computerized adaptive test of vocabulary three times to assess the verbal intelligence of chatGPT (GPT 3.5) and Bing (based on GPT 4). There was no difference between their performance; both performed at a high level, outperforming approximately 95% of humans and scoring above the level of native speakers with a doctoral degree. In 42% of test items that were administered more than once these large language models provided different answers to the same question in different sessions. They never engaged in guessing, but provided hallucinations: answers that were not among the options. Such hallucinations were not triggered by the inability to answer correctly as the same questions evoked correct answers in other sessions. The results implicate that psychometric tools developed for humans have limitations when assessing AI, but they also imply that computerised adaptive testing of verbal ability is an appropriate tool to critically evaluate the performance of large language models.

## Introduction

Large language models (LLMs) like OPEN AI’s ChatGPT provide a revolutionary development in AI, imitating human-like capacities in creating various kinds of content from essays to computer code. In order to evaluate the performance of an LLM and to compare the performance of different LLMs with one another as well as with human performance a quantitative assessment is required. However, classic comparisons of human and artificial intelligence, like the Turing test, typically do not differentiate human performance; they evaluate AI with respect to their ability to imitate *universal* aspects of human cognition, like the ability to communicate using language. Yet the focus of the psychology of human intelligence is on *individual differences*; IQ itself is a relative indicator comparing one’s performance to one’s peers [1].

A subfield of AI does engage in using tests to measure machine intelligence and there have been several attempts to evaluate the performance of AI by having it complete various tests of intelligence designed for humans [2]. In fact, it has been claimed that the best measure of the actual intelligence of AI is how well it can perform on psychometric tests designed for humans [3]. Others argue that this approach is completely misleading [4]. Alternatively, a new, universal test of computational complexity is offered to evaluate the capabilities of AI systems, independent of the content of the actual task which they perform [5, 6].

We administered a computerized adaptive test of vocabulary to two versions of the same LLM. We chose a psychometric test of vocabulary to evaluate the performance of LLMs for two reasons. First, while universal tests are ambitious, domain-specificity seems to be a strong characteristic of both human and artificial intelligence [7]. Therefore, while psychometric tests might indeed not be valid indicators of the intelligence of AI systems designed to process completely different kinds of material, verbal ability appears to be a valid indicator of individual differences between *language-specific* AI models.

Second, vocabulary is a central indicator of human intelligence–so much that it has been argued that “vocabulary is probably the best single indicator of a person’s level of intelligence” [8]. While this claim might be controversial, it is indeed the case that all major batteries designed to measure human cognitive abilities, like the Wechsler Adult Intelligence Scale [9] or the Woodcock-Johnson Tests of Cognitive Abilities [10] include a test of vocabulary.

The currently most accepted model of the structure of human cognitive abilities, the Cattell-Horn-Carroll model [11, 12] differentiates between broad and narrow cognitive abilities in a hierarchical fashion, so that narrow abilities are represented at a lower stratum under their corresponding broad ability. In this model, vocabulary is part of the narrow ability Lexical knowledge (VL): “Knowledge of the definitions of words and the concepts that underlie them; vocabulary knowledge” [12]. Lexical knowledge, in turn, is represented under the broad ability Comprehension-knowledge (Gc). In earlier ability models this ability was referred to as crystallized intelligence [13, 14]. Overall, not only is vocabulary an ecologically valid measure of the intelligence of LLMs, it in fact taps on a central ability in the structure of the specific abilities that constitute human intelligence.

## Methods

We decided to use a computerized adaptive test (CAT) [15]. While in traditional tests all examinees are administered the same, fixed set of items, under CAT an algorithm selects individual items from an item bank so that item difficulty is always close to the actual ability estimate based on all previous answers. Therefore, most of the items administered during a CAT session meet the examinee’s ability, which allows for CAT to be a more precise mode of assessment than traditional tests consisting of fixed items.

CAT also allows for setting individual characteristics of the algorithm, one of which is the randomization of item selection. When randomization is low, the selection is almost deterministic: the item closest in difficulty to the current ability estimate is selected. When randomization is high, an item is randomly selected from a set of items all of which are close in difficulty to the examinee’s ability. Varying the randomization parameter on repeated testing occasions allows for the LLMs to take a different, yet overlapping set of items.

In a non-adaptive, fixed item test only the examinee’s total score is compared to the total score distribution of a norm population, hence items do not differentially contribute to the ability estimate. Additionally, a mistake on any item necessarily results in a decrease in the total score, regardless of answers to other items. Under CAT, however, the algorithm allows to compensate for a mistake early on in the process. This is because CAT is based on item response theory [16] and as such the individual answers do not equally contribute to the estimated level of ability. This estimate indicates the likeliest level of ability at which the given pattern of *all previous answers* might have occurred. Therefore, correctly answering difficult items can “compensate” for failing easy ones.

Most importantly, CAT is the optimal testing strategy if we have no advance estimate of the examinee’s ability range. In traditional, non-adaptive tests the number of items is fixed, hence such tests must target a specific range in the ability distribution. When testing humans, the selection of an appropriate test is guided by the characteristics of the examinees, such as age or highest level of education, which allow for an approximation of the examinees’ ability range. For instance, different versions of the Raven’s Progressive Matrices (Coloured, Standard, Advanced) target examinees with different age and ability. In CAT, however, the number of items in the item bank can cover the entire distribution of human ability. Since we have no advance information about the vocabulary of LLMs, or of the cognitive ability of AI in general, CAT is the best solution for evaluating their performance in comparison to humans.

We administered 20 items of NoVo (Non-Directional Vocabulary Test), a computerised adaptive test measuring vocabulary. NoVo has a novel item format: instead of having to select the synonym of a target word from eight options, like in the case of fixed item tests such as the Mill Hill Vocabulary Scale (MHVS), examinees have to select two out of nine words that are closest in meaning (for human administration the words are arranged in a 3x3 matrix). This is the same format as in SAM (Scrambled Adaptive Matrices) [17], a nonverbal test of fluid reasoning, in which examinees have to select two elements arranged in a 3x3 matrix so that if the selected elements are switched the matrix is complete in a logical fashion. This is different from traditional matrix tests, like the Raven’s Progressive Matrices (RPM) in which examinees have to complete a 3x3 matrix by selecting the missing element from eight options. In fact, NoVo is complimentary to SAM the same way as the MHVS was developed to compliment the RPM [18]. The psychometric parametrization of items as well as the estimation of latent ability (θ) scores followed the 2-parameter logistic item response theory model [19].

The following is an example item from NoVo as administered to LLMs: “*Which two words are most similar to each-other in meaning out of the following words*: *begin*, *believe*, *add*, *buy*, *stop*, *start*, *design*, *read*, *spend*?”

In order for the testing session to be adaptive each answer provided by the LLM was entered manually to the adaptive algorithm, which selected the next item based on the answer. The CAT stopping rule was based on fixed testing length: the session stopped after administering 20 items. We administered NoVo three times to chatGPT and Bing each. At the given date chatGPT was running on version 3.5 while BingChat was based on GPT-4,. On the first occasion, randomization was high, on the second occasion it was low, while on the third occasion it was medium. As a result, there were overlapping items in the individual occasions: overall, ChatGPT and Bing were administered 35 and 41 individual items of 60 total items, respectively. This allowed to investigate whether LLMs would provide different answers on different occasions.

Parameters of both LLMs–like temperature or top_p–were used at their default setting. When responding to a question, LLMs create a pool of tokens where each token has a probability of being adequate. The parameter top_p refers to the probability threshold for a token to be selected into the pool of tokens from which the LLM selects; if top_p is low then only probable answers become part of the pool. Temperature refers to the chance of actually selecting a less probable token from the pool of tokens; when temperature is 0 the LLM will always select the most probable token. Overall, top_p and temperature determine the “creative aspect” of chatGPT’s behavior. In our study, we have not changed the default values in order to emulate the in typical experience a user has with LLMs. For the same reason, we have used Bing chat in its default setting (‘Balanced’).

## Results

### Comparison with human performance

The performance of ChatGPT and Bing was compared to human performance on the basis of a large, non-representative sample (N = 9093), where respondents indicated their highest level of education and whether they were native speakers of English. Table 1 summarizes the average standing of each educational subsample compared to the total sample of test-takers.

**Table 1:** NoVo normative data as the function of native vs. non-native and educational stratification. The mean of each subsample is compared to the mean of the total sample; the values are z scores, therefore 0 represents the mean of the total sample and the values are in standard deviation (SD) units calculated on the total sample.

Education	Non-Native Mean	Native Mean
Attending elementary school	-0.56	-0.1
Completed elementary school	-0.18	0.41
High school	-0.09	0.59
College diploma	-0.02	0.63
University diploma	0.14	0.85
PHD or equivalent	0.2	1.23

The results were calculated according to item response theory, ability estimates are therefore provided as θ scores [16, 20]. In item response theory, θ represents the latent construct of interest–in this particular instance, verbal intelligence–that is estimated based on the string of correct and incorrect responses provided by the examinee. Technically, θ is the point in the ability scale where the given string of responses is most likely to have occurred. θ is a standardized z-score: it compares test-takers to the norming sample and expresses performance in standard deviation units; if θ = 0 then the test-taker performs at the average of the norming sample, if θ = 1 then the test-taker performs 1 standard deviation above the mean, etc. Since θ scores are normally distributed it is possible to convert θ to percentile (%ile), indicating the percentage of the norming sample that the test-taker has outperformed.

**Fig 1:**
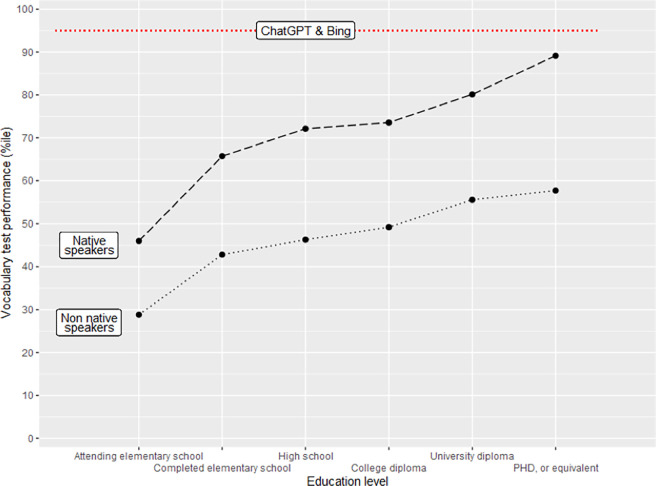
The performance of the LLMs compared to human performance as the function of educational stratification.

Overall results are presented in Table 2. Both chatGPT and Bing performed at a very high level compared to humans: they outperformed approximately 95% (92–96%) of the normative sample. Even more revealing is the result that compares the performance of LLMs to humans as the function of educational stratification (Fig 1): both LLMs outperformed the mean of native speakers who hold a PhD degree. The performance of the LLMs was also uniform across sessions: the differences are minimal and within error margin. Interestingly, Bing, which is based on GPT4, did not outperform chatGPT 3.5.

**Table 2:** The test results of ChatGPT and Bing on the individual occasions. The results are expressed as 1) thetas as customary in item response theory and 2) as percentile scores. Thetas are Z-scores, i.e., they are expressed in standard deviation units, while percentile scores indicate the percentage of the normative sample that is outperformed.

LLM	Occasion	Randomisation	Result (θ)	Result (%ile)
ChatGPT (3.5)	1^st^	High	1.52	94.
ChatGPT (3.5)	2^nd^	Low	1.6	95.
ChatGPT (3.5)	3^rd^	Mid	1.7	96.
Bing (4)	1^st^	High	1.4	92.
Bing (4)	2^nd^	Low	1.6	95.
Bing (4)	3^rd^	Mid	1.71	96.

### Test-taking characteristics of large language models

**Fig 2:**
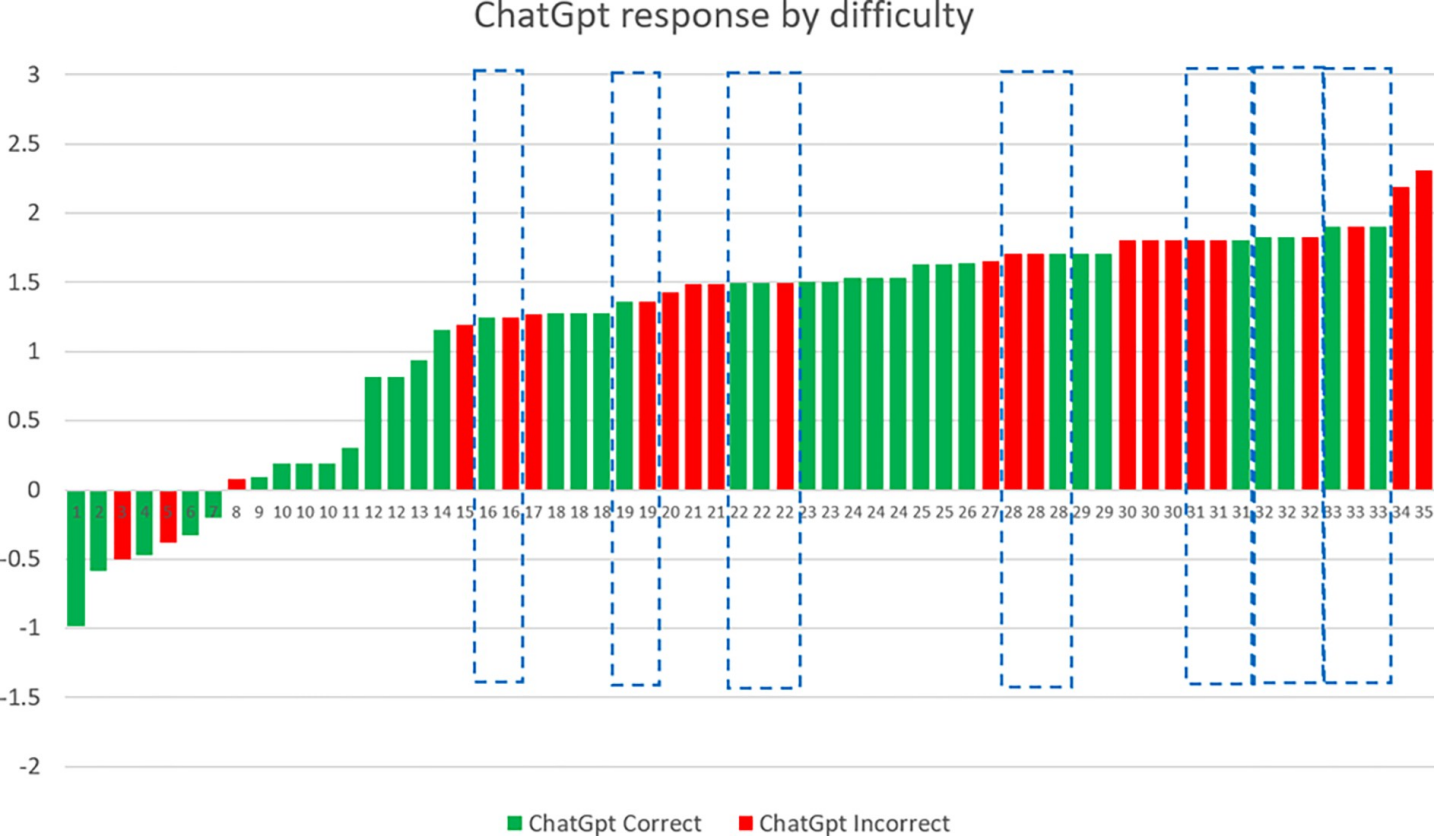
Responses of ChatGPT (3.5) as the function of item difficulty. Correct answers are green, mistakes are red. Highlighted are cases where the LLM gave both correct and incorrect answers to the same item on different occasions.

**Fig 3:**
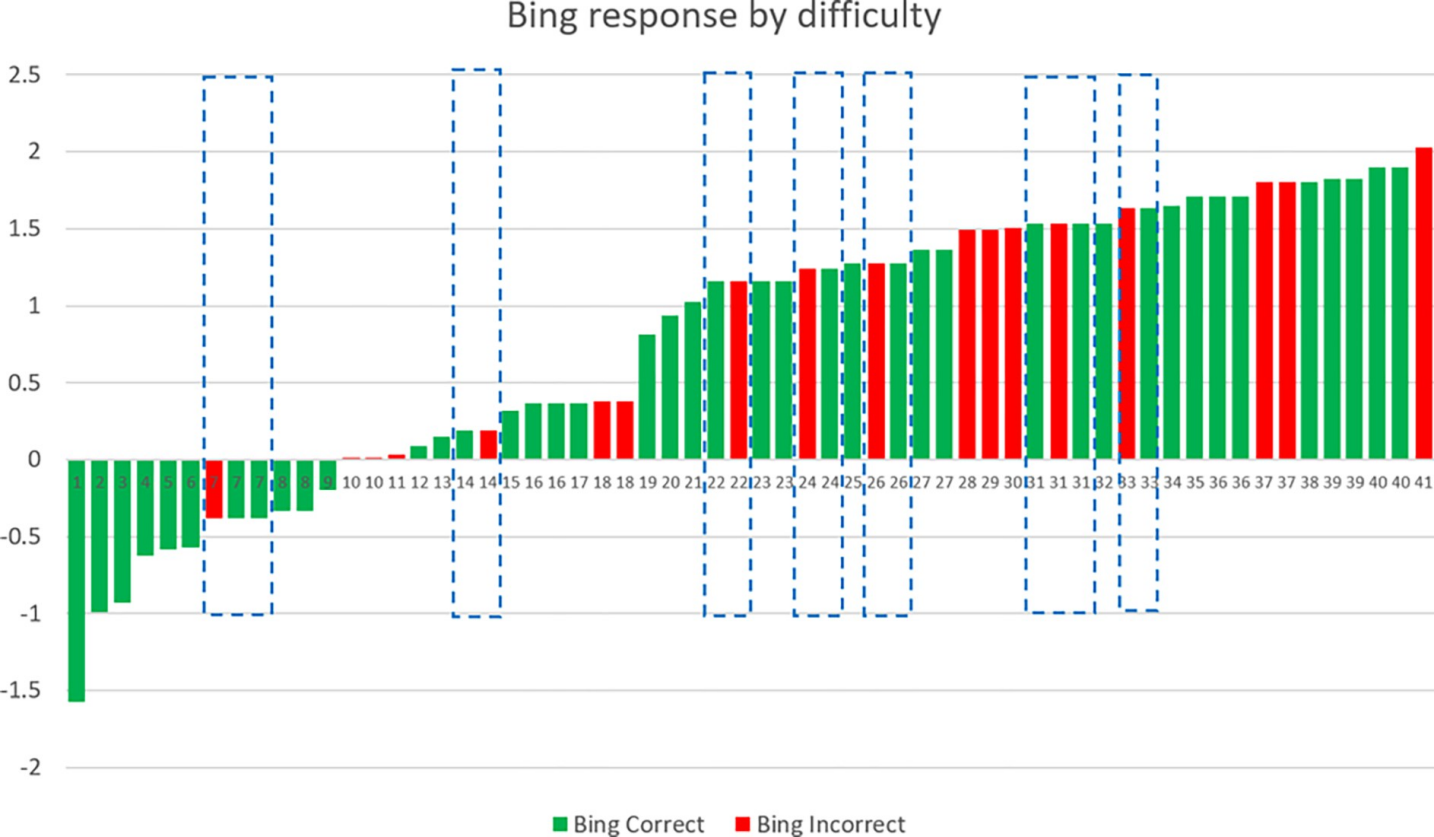
Responses of Bing (based on 4.0) as the function of item difficulty. Correct answers are green, mistakes are red. Highlighted are cases where the LLM gave both correct and incorrect answers to the same item on different occasions.

The analysis of answers to individual items allows for the comparison of performance as the function of item difficulty in humans and LLMs. Figs 2 and 3 show items with increasing difficulty; difficulty parameters (depicted on the Y axis) were, of course, obtained by an IRT analysis of *human* performance. In item response theory difficulty is represented on the same scale as ability, so that the difficulty of an item is equal to the ability level at which the probability of solving the item is 50%. For instance, in the case of item 22 on Fig 2, humans whose ability is 1.5 standard deviations above the mean have a 50% probability of arriving at a correct solution. Columns in red indicate that the LLM provided an incorrect answer.

Because the randomized adaptive test administration allowed for overlapping items, we could investigate the consistency of responses to repeatedly presented items. In Figs 2 and 3 identical numbers indicate identical items; cases where answers were incongruent are highlighted. 16 and 17 items occurred at least twice for ChatGPT and Bing, respectively, and there were 7 instances for both ChatGPT and Bing when they provided different answers to the same item. This means that in 42.42% of repeated item administrations (i.e, in 14 out of 33 occasions) the LLMs gave different answers to the same question in different sessions.

An analysis of errors made by LLMs indicates that the ways these models fail to find the synonymous pair out of the nine words are not identical to how humans make mistakes:

1. The LLM did not find any synonyms from the nine options (e.g. “I’m sorry but none of the given pairs of words are synonyms.”). This type of error was infrequent. The LLM always frames its response in an objective manner (none of the pairs are synonyms) instead of subjective (I was unable to find a pair).
2. The LLM did not correctly evaluate the “distances in meaning” between pairs of words (e.g. to the item: “*Which two words are most similar to each-other in meaning out of the following words*: *beauty*, *spell*, *Talisman*, *marathon*, *Dome*, *mountain*, *chasm*, *gorge*?*”* the answer was: “*Out of the given words*, *“beauty” and “spell” are most similar in meaning as they both refer to something that is attractive or pleasing to the senses*. *“Talisman” refers to an object that is believed to have magical powers and bring good luck*, *while “marathon” refers to a long-distance running race*. *“Dome” refers to a rounded vault forming the roof of a building or structure*, *while “mountain” refers to a large natural elevation of the earth’s surface rising abruptly from the surrounding level; an overview is a general summary or survey of something*. *Finally*, *“chasm” and “gorge” both refer to a deep fissure in the earth’s surface*.*”)*
3. The LLM “hallucinated”, i.e. it gave an answer that was not listed among the options (e.g. to the item “*Which two words are most similar to each other in meaning out of the following words*: *local*, *final*, *old*, *high*, *important*, *last*, *large*, *long*, *different*?*”* the answer was: *“The two words that are most similar in meaning are large and big*. *Large refers to of considerable or relatively great size*, *extent*, *or capacity*. *Big refers to of considerable size or extent*.*”)*.

Of these three mistakes, the most intriguing and most unique to LLMs are hallucinations, a recurring problem in LLM models [21, 22]. In most instances, hallucination means that the LLM fabricates factual information, such as fake references to scientific publications or fabricated biographical information. In our case it involved defying the task instruction and not selecting from the options provided.

## Discussion

There seems to be a rather high inconsistency in the answers provided by both engines: in approximately 42% of test items that were administered more than once LLMs provided different answers to the same question. Such a high level of intra-individual variability in unlike human test-takers where variation is expected to manifest itself primarily between subjects who, in turn, are expected to perform in a consistent way. In psychometrics it is assumed that the actual test score is the composite of a latent *true score* (the signal) and *measurement error* (the noise). If in the case of AI the true performance itself contains a substantial amount of noise this calls into question the interpretation of standard psychometric constructs, such as psychometric reliability, which is defined as the ratio of true score variance and total variance, i.e. how error-free the measurement is.

Similarly remarkable were hallucinations, where the LLMs gave an answer that was not among the options. Moreover, in many instances a hallucination followed a correct answer to the same question in a previous session, indicating that hallucinations were not triggered by the inability to answer correctly. The mode of answering is different from that of humans in other ways, too. A lack of information about the meaning of one or more words from the list–the most typical source of error in the case of humans–did not seem to occur in Bing or ChatGPT.

In order for standardized psychometric tests to be valid indicators of AI performance, the understanding of how AI performs on these items and why their test-taking behavior is different from humans is necessary. Otherwise, it is quite possible that the same items measure different traits in machines and humans; that is, the psychometric concept of *measurement invariance* (MI) across machines and humans is violated. Measurement invariance is a statistical property of tests and items that indicates that the same construct is being measured across different groups [23, 24]. That is, a test item is invariant if members of the different groups who have the same standing on the construct being measured have an equal probability of arriving at a correct answer on the item. Items for which measurement invariance is violated, i.e. for which the probability of answering correctly depends on group membership above and beyond one’s standing on the construct being measured, are considered biased and do not allow for a fair comparison of members of the groups in question. There are statistical methods to investigate such item bias, primarily *differential item functioning*, but they require substantial statistical power, hence in order to establish DIF for LLMs and humans the assessment of several hundred LLMs will be required [25].

Overall, both LLMs performed at a high level: they outperformed 19 out of 20 humans and scored above the mean level of native speakers who hold a doctoral degree. In the light of our results these LLMs seem to have a high verbal ability, in fact, were they humans, they would be probably identified as gifted. Moreover, there does not seem to be much room for improvement for LLMs on ability tests normed on human populations: in the foreseeable future, AI is likely to outperform 100 out of 100 humans in vocabulary. This means that a ceiling effect is likely to occur in AI for psychometric tests designed for humans. This tendency would eventually make it necessary to develop items of superhuman difficulty that can serve for the meaningful comparison of LLMs or other kinds of AI. For the nearer future, when trying to tell a machine from a human in a chat, too sophisticated, rather than too shallow communication could be a reason for suspicion.
